# Photosensitizers for Photodynamic Therapy of Brain Cancers—A Review

**DOI:** 10.3390/brainsci13091299

**Published:** 2023-09-08

**Authors:** Dorota Bartusik-Aebisher, Paweł Woźnicki, Klaudia Dynarowicz, David Aebisher

**Affiliations:** 1 Department of Biochemistry and General Chemistry, Medical College of the University of Rzeszów, 35-959 Rzeszów, Poland; dbartusikaebisher@ur.edu.pl; 2 Students English Division Science Club, Medical College of the University of Rzeszów, 35-959 Rzeszów, Poland; pw118616@stud.ur.edu.pl; 3 Center for Innovative Research in Medical and Natural Sciences, Medical College of the University of Rzeszów, 35-310 Rzeszów, Poland; kdynarowicz@ur.edu.pl; 4 Department of Photomedicine and Physical Chemistry, Medical College of the University of Rzeszów, 35-959 Rzeszów, Poland

**Keywords:** brain cancers, photodynamic therapy, molecular targeted therapies, photosensitizers

## Abstract

On average, there are about 300,000 new cases of brain cancer each year. Studies have shown that brain and central nervous system tumors are among the top ten causes of death. Due to the extent of this problem and the percentage of patients suffering from brain tumors, innovative therapeutic treatment methods are constantly being sought. One such innovative therapeutic method is photodynamic therapy (PDT). Photodynamic therapy is an alternative and unique technique widely used in dermatology and other fields of medicine for the treatment of oncological and nononcological lesions. Photodynamic therapy consists of the destruction of cancer cells and inducing inflammatory changes by using laser light of a specific wavelength in combination with the application of a photosensitizer. The most commonly used photosensitizers include 5-aminolevulinic acid for the enzymatic generation of protoporphyrin IX, Temoporfin—THPC, Photofrin, Hypericin and Talaporfin. This paper reviews the photosensitizers commonly used in photodynamic therapy for brain tumors. An overview of all three generations of photosensitizers is presented. Along with an indication of the limitations of the treatment of brain tumors, intraoperative photodynamic therapy and its possibilities are described as an alternative therapeutic method.

## 1. Introduction

According to Global Cancer Statistics 2020, 308,102 new cases of brain and central nervous system cancer have been diagnosed worldwide [1]. Studies have shown that brain and central nervous system tumors are among the top ten causes of death. Due to the scale of this problem and the percentage of patients suffering from brain tumors (especially glioblastoma), new innovative therapeutic methods are constantly being sought [2]. The most common brain tumors are gliomas, which account for about 78% of all brain tumors. In addition, they are the most common primary malignant tumors of the central nervous system [3]. According to the classification of the World Health Organization (WHO), gliomas are classified on a scale from I to IV depending on the degree of malignancy. Grade I and II gliomas are considered low-grade tumors, which include astrocytomas and oligodendrogliomas. Grade III and IV gliomas are high-grade malignancies, which include anaplastic astrocytomas and glioblastomas [4]. A small proportion of gliomas are caused by congenital disorders such as neurofibromatosis or tuberous sclerosis [5]. Although they are relatively rare, they are all characterized by rapid growth, angiogenesis and infiltration into adjacent tissues, which significantly hinders the complete resection of the tumor [6]. In the brain, the blood–brain and blood–tumor barriers limit the possibility of metastasis, but at the same time significantly hinder the treatment of these tumors. The basic therapeutic variants of this type of cancer are surgical resection, adjuvant radiotherapy and chemotherapy. Malignant gliomas are characterized by a large central necrosis surrounded by a group of invasive cells that migrate beyond therapeutic margins and contribute to tumor recurrence [7]. Recurrence of the disease is very common and occurs in almost 90% of patients [8]. Therefore, new therapeutic variants of brain tumors are still being sought [9].

**Figure 1:**
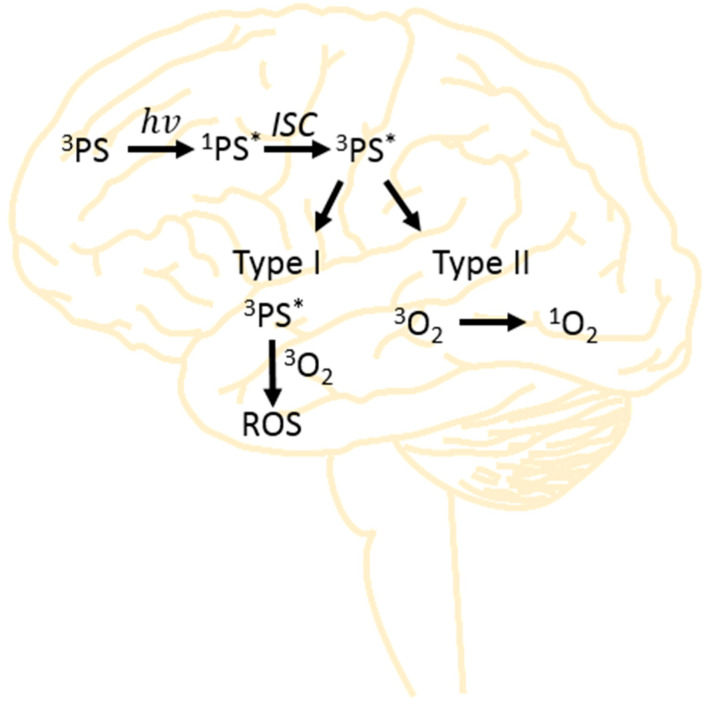
Scheme of Type I and Type II reaction pathways that occur during photodynamic therapy.

Photodynamic therapy (PDT) is one of the alternative methods of treating various types of cancer [10] including brain tumors [11,12] and other diseases (i.e., inflammation [13], bacterial infections [14] and dermatological diseases [15]). A significant increase in the widespread use of PDT occurred at the beginning of the 21st century [16]. The basis of the action of PDT is an interaction of three substrates: a photosensitizer (PS), oxygen and light [17]. Photosensitizers are excited to a higher energy level by absorption of a photon and subsequently form a relatively long-lived triplet state. The triplet state can either transfer energy to triplet oxygen forming singlet oxygen (Type II) or initiate the generation of other free radical reactive oxygen species (ROS) (Type I) [18] (Figure 1).

In photodynamic therapy, two pathways of cytotoxic ROS product generation responsible for the destruction of cancer cells are distinguished [19]. In Type I, when the PS is irradiated, the ground-state PS (^3^PS) absorbs energy and is converted to a singlet state (^1^PS*, *-excited state). Through intersystem crossing (ISC), the excited state (^1^PS*) can relax to the triplet-state (^3^PS*) manifold [20]. Thus, the PS in the triplet state (^3^PS*) can undergo electron transfer with substrates, i.e., oxygen. The reactive oxygen species (ROS) produced include hydrogen peroxide, superoxide anions and hydroxyl radicals, which cause specific cellular damage and contribute to radical reactions [21,22]. On the other hand, in a Type II process, the energy from the PS triplet (^3^PS*) is transferred to triplet oxygen (^3^O_2_), forming cytotoxic singlet oxygen (^1^O_2_). This type of oxygen specifically interacts with various components of the cell, initiating cell death [23]. Both Type I and Type II processes can occur simultaneously with one process dominating depending on the type of photosensitizer and its concentration. Therefore, the purpose of this review is to characterize the most commonly applied and used photosensitizers in photodynamic therapy for the treatment of brain tumors.

## 2. Materials and Methods

**Figure 2:**
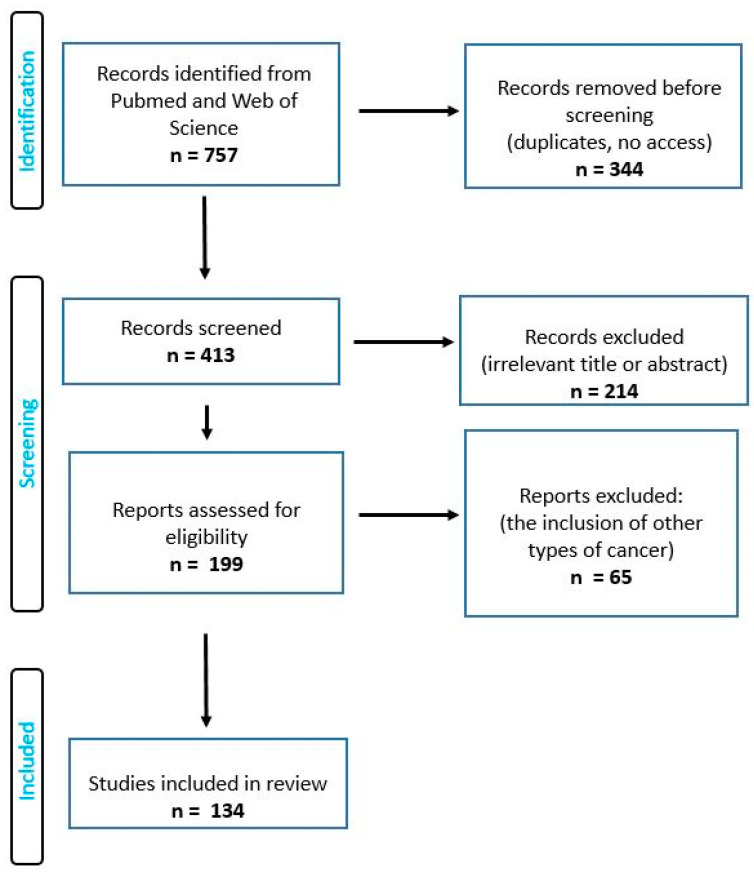
PRISMA flow diagram of the studies included.

A search that focused on the types of photosensitizers used in photodynamic therapy for the treatment of brain cancers was conducted by using articles from PubMed, ScienceDirect, Web of Science and Google Scholar from 1990 to June 2023. The search term included the phrase “photosensitizers in photodynamic therapy of brain cancer”. The authors of this review worked on the basis of an agreed scheme, selecting articles based on their title, language, abstract and access. Duplicate records were removed. This review was conducted based on the Preferred Reporting Items for Systematic Reviews and Meta-Analyses (PRISMA) guidelines [24]. Full-text and accessible articles were reviewed. In order to minimize the selection bias, the inclusion and exclusion criteria (Table 1) were established as follows: 

This review included only cases with brain tumors in which both in vivo and in vitro studies were qualified. Both review and research articles were included, and this review included studies with adult patients. This review included papers that characterized such photosensitizers as 5-ALA, Temoporfin, Photofrin, Hypericin or Talaporfin, with the latest works describing other types of photosensitizers; additionally, photosensitizers of the third generation were also included. The following were excluded from this review: papers written in a language other than English or Polish; articles presenting PDT for other types of cancer; studies using chemotherapy in combination with PDT; studies using magnetic resonance imaging, computed tomography or other tools; and studies on pregnant women and children. Figure 2 shows PRISMA flow diagram of the studies included.

## 3. A Review of the Literature

### 3.1. Application of Photodynamic Therapy

Photodynamic therapy is one of the therapeutic methods used to treat brain tumors. Due to its low invasiveness and effectiveness with other diseases, it is also more and more often practiced in the treatment of neurological diseases, including brain tumors [25]. The main principle of PDT operation is the application of a photosensitizer (in various forms) and then exposure to laser light (hv), in which the wavelength is correlated with the photosensitizer used [26]. Under the influence of light, the photosensitizer changes from the ground state to the excited singlet state (^1^PS*), which is converted to the excited triplet state (^3^PS*) by intersystem crossing (ISC). The described transition generates two types of reactions [27]. In a Type I reaction, the photosensitizer in an excited state interacts with fatty acids found in the lipids of cell membranes [28]. Then, an electron or a proton is transferred, resulting in the formation of organic radicals. Combined with cellular oxygen, they can generate reactive oxygen species (ROS) [29]. In turn, the Type II reaction consists of energy transfer, which in turn leads to the formation of singlet oxygen (^1^O_2_) [30]. Both types of reactions can occur simultaneously. Their severity depends on the type of photosensitizer used, its dose and physical and chemical properties. As a result of both types of reactions, the cancer cells are destroyed in the treated tumor area [31].

The method of application of the therapy (both the photosensitizer and the method of delivering the light) varies and depends on the type of disease [32]. In dermatological cases (all kinds of skin inflammatory changes, cancer or other diseases), the photosensitizer is applied topically to the skin [33]. Laser light is also delivered locally and centrally to the lesions.

In dermatological cases, the most commonly used photosensitizer is 5-ALA and methyl aminolevulinate (MAL) [34]. When applying 5-ALA, lasers with a wavelength of 630–650 nm are used. In the case of MAL applications, red light in the range of 570–670 nm is applied [35]. 

In the case of brain tumors, one of the newest therapies is intraoperative photodynamic therapy [36].

### 3.2. Photosensitizer

#### 3.2.1. 5-Aminolevulinic Acid

5-aminolevulinic acid (5-ALA) is the precursor of the photosensitizing compound protoporphyrin IX (PpIX). Cells generate PpIX from 5-ALA through the heme biosynthetic pathway. The use of 5-ALA allows for selective tumor targeting due to the intracellular metabolism of this compound [37]. The exact reasons for this phenomenon are unknown; however, it is believed that PpIX is selectively accumulated in cancer cells due to the specific regulation of their heme enzymes [38,39,40,41]. This feature made 5-ALA a new standard in the surgical treatment of malignant gliomas. In addition, the high tumor selectivity of fluorescent PpIX accumulation enables intraoperative fluorescence guidance that is unaffected by brain displacement. Thanks to this, the complete neurosurgical resection of the growing tumor is simpler, which improves the prognosis of patients [42,43]. The only undesirable effect of this action is minimal skin sensitization caused by the administration of 5-ALA [43]. 

Surgical resection guided by photosensitizer fluorescence followed by photodynamic therapy (PDT) has been observed to prolong the mean survival in patients with glioblastoma [44]. In addition, in vivo studies have confirmed that 5-ALA-PDT can be an effective method of treating gliomas by inhibiting tumor growth [45]. Light irradiation (635 nm) of malignant glioma cells containing PpIX after pretreatment with 5-ALA causes their death by various pathways, including apoptosis and necrosis [45,46,47,48]. The basic mechanism of cell death induced by 5-ALA-PDT in glioblastoma cells has not been fully elucidated and is still a subject of controversy [45,49]. Some studies suggest that 5-ALA-PDT intensifies cell necrosis, which may result from the destruction of tumor microvessels [45,46]. Others claim that this therapy induces massive apoptosis by direct activation of the mitochondrial pathway, disruption of mitochondrial membrane potential function and release of cytochrome c. These findings suggest that 5-ALA-PDT is a promising therapy for the treatment of apoptosis-resistant malignant gliomas [49]. Glioblastoma cell death was common due to necrosis when assessed 18 h after PDT. PDT combined with 5-ALA promotes the death of necrotic cells, and is dose-dependent [49,50]. Another 5-ALA-PDT study showed large areas of central tumor necrosis, although clusters of viable tumor cells were often located on its periphery [51]. It has been demonstrated that the inhibition of the nuclear factor NF-κB increases the death of glioma cells in response to 5-ALA-PDT, which makes the tumor more sensitive to therapy [52].

The results of another study indicated that PDT repeated at relatively long intervals (weeks) was more effective in inhibiting the growth of brain tumor spheroids than daily fractionated PDT or a single treatment. Preliminary evidence for the increased efficacy of repetitive PDT and low-frequency fluency treatment has been reported [53]. Another study showed that although treatment with a low fluence rate was better tolerated, 5-ALA-PDT induced more severe tumor necrosis when using fractionated delivery at a high fluence rate [54]. Due to the discrepancy in results, further research is needed to determine the most effective 5-ALA-PDT dose. This therapy can be used in the treatment of resistant malignant gliomas as it has the ability to eliminate the stem cells responsible for tumor insensitivity to radio and chemotherapy [55]. 5-Aminolevulinic acid-PDT has been shown to sensitize human glioblastoma cells to RIP3 (Receptor-Interacting Protein 3)-dependent cell death [56]. Putting the patient into hypothermia for the duration of the procedure seems to be potentially important when conducting photodynamic therapy with the use of 5-ALA. Studies on rats subjected to mild hypothermia showed a five-fold increase in PpIX fluorescence in tumors, as well as almost complete cellular protection in normal brain structures [57]. In the case of 5-ALA-PDT therapy for meningioma, it was observed that ciprofloxacin and a longer incubation time of 5-ALA significantly increased the cytotoxic effect of PDT. Since this antibiotic is a widely used agent with good tissue penetration, low toxicity and a favorable risk profile, it is important to undertake further research [58]. Standalone interstitial photodynamic therapy (iPDT) (i.e., without combined craniotomy and intrahilar PDT) in the treatment of brain tumors with the use of 5-ALA seems to be the best option in terms of side effect control as it avoids the occurrence of permanent neurological deficits while reducing the risk of hemorrhage and sepsis [59].

5-Aminolevulinic acid administration resulted in a 2.5-fold increase in PpIX accumulation in the cerebral cortex of mice compared to untreated mice. A histological examination showed damage to some neurons and cortical vessels. 5-Aminolevulinic acid-PDT selectively changes the expression of the proteins involved in the epigenetic regulation of transcription, histone modification, DNA repair, nuclear protein import and proliferation, which indicates the presence of epigenetic markers of photooxidative damage to normal brain tissue [60]. 5-Aminolevulinic acid-mediated PDT has been shown to be safe at doses of 90 mg/kg or less followed by 100 J/cm^2^ light irradiation in rat brains. It was observed that a dose above this threshold led to irreversible damage to regions of the blood–brain barrier (BBB) and the brain itself. In healthy rat brain tissue, neurological signs developed after the administration of 5-ALA at a dose of 240 or 120 mg/kg with concomitant irradiation of 100 or 400 J/cm^2^. Breakdown (BBB) occurred at 90 mg/kg and 100 J/cm^2^. The number of neurons decreased at the dose of 200 mg/kg and 400 J/cm^2^, respectively [61]. Fluorescence microscopy of frozen rat brain tumor tissue sections showed that the photosensitizer content was limited and variable in the tumor tissue entering the normal brain. 5-Aminolevulinic acid-PDT with high doses of light caused significant damage to both the tumor and normal brain tissue [62].

It has been shown that the activity of the 5-ATP-binding cassette transporter ABCG2 may affect the effectiveness of PDT by regulating the accumulation of porphyrins in cancer cells. In response to the photoreaction of porphyrins leading to oxidative stress, the nuclear factor erythroid-derived 2-related transcription factor can transcriptionally increase the level of ABCG2 [63]. Glioblastoma cells with high ABCG2 expression accumulate less photosensitizers and require higher doses of light for elimination. Elevated levels of ABCG2 in doxycycline-induced sU251MG-V glioblastoma cells led to a reduced accumulation of PpIX, and higher doses of light were required to reduce cell viability. By inhibiting the ABCG2 transporter with the effective and nontoxic ABCG2 inhibitor KO143, the accumulation of PpIX and the effectiveness of PDT could be significantly increased [64]. The inhibition of ABCG2, e.g., through a strong ABCG2-inhibitor gefitinib, restores the full susceptibility of cancer cells to photodynamic treatment [64,65]. Nitric oxide (NO) has been found to play a key role in various manifestations of the increased aggressiveness exhibited by 5-ALA-PDT-resistant cells. Studies have shown that endogenous NO in various mouse tumor models significantly reduces the effectiveness of PDT. Nitric oxide produced by photostressed cancer cells may induce anti-PDT effects, as well as intensify their proliferation and migration [66].

#### 3.2.2. Temoporfin—THPC

Temoporfin (5,10,15,20-tetra(*m*-hydroxyphenyl)chlorin) is the active ingredient of Foscan^®^, which is authorized in the EU for photodynamic therapy of head and neck squamous cell carcinoma (HNSCC) [67,68,69]. This compound seems to be the most promising photosensitizer in the treatment of brain tumors [70,71]. Since Temoporfin causes complaints associated with high photosensitivity of the skin, in order to avoid them, an intratumoral route of administration of *m*-THPC was developed. A study showed that intratumoral administration of the drug has comparable results to the systemic route in terms of intracellular uptake efficiency and the tumor-to-normal-tissue ratio, with the advantage of a much shorter time to reach the optimal intracellular concentration (four hours after an injection of *m*-THPC) [70]. Preliminary studies have confirmed that bPDT using *m*-THPC can kill Grade 4 astrocytoma cells transfected with luciferase in vitro [72]. Research was also undertaken on the combination of *m*-THPC with gold nanoparticles (AuNPs), and it was observed that under the influence of laser radiation of the PDT/PTT combination, phototoxicity was twice as high as compared to treatment with only PDT or only PTT [73].

#### 3.2.3. Photofrin

Photofrin^®^ is a photosensitizing compound that is effective in the treatment of brain tumors. It has been shown that low-dose subcytotoxic PDT with Photofrin^®^ significantly inhibits the invasiveness of U87 and U25ln glioblastoma cells [74]. Case studies of 112 patients with malignant gliomas, metastatic brain tumors and meningiomas treated with Photofrin^®^ PDT were reviewed. The overall survival after PDT of 96 patients with epidural gliomas was 42 weeks, and the 1- and 2-year survival was 40% and 22%, respectively. No significant postoperative complications were observed in 75% of the patients. Photofrin^®^-PDT was found to be safe. It is assumed that higher doses of light than those used in the described patients may provide better effectiveness of the therapy [75]. 

Photofrin^®^-PDT can shrink the tumor, especially at high light doses. It also has the ability to induce VEGF expression in tumor-adjacent tissue (BAT). This is associated with tumor recurrence; therefore, it is believed that PDT in combination with antiangiogenic agents may be an effective strategy for the treatment of glioblastoma [76]. In a study that measured the response of normal brain and U87 human glioma implanted in rat brains to photodynamic therapy (PDT) by using Photofrin^®^ as a photosensitizer, the U87 human brain tumor model and normal athymic rat brain were found to be sensitive to PDT and Photofrin^®^ with a dose-dependent optical response [77]. Photofrin^®^-PDT has been shown to cause a transient increase in cell proliferation associated with the strong activation of astrocytes and microglia in the treated region, without causing significant cell death. The results of one of the studies indicate that subclinical photodynamic therapy using Photofrin^®^ locally changes brain homeostasis without significant disturbances in the tissue architecture [78]. It has been observed that the use of Photofrin^®^ encapsulated in liposomes significantly increases photosensitizer uptake by the tumor, as well as its destruction in relation to Photofrin^®^ in dextrose. At the same time, there was no difference in tissue destruction in the uninjured brain with or without the liposome carrier. The results of this study suggest that the liposomal carrier enhances the therapeutic efficacy of PDT in the treatment of 9L tumors [79,80]. Adjuvant repetitive PDT seems to provide local control of metastatic brain cancers, with the best results obtained in lung cancer [81]. The effectiveness of Photofrin^®^-PDT can be improved by administering buthionine sulfoximine, which reduces the level of glutathione, which in turn is responsible for ROS inhibition [82]. Tamoxifen (TMX), a protein kinase C (PKC) inhibitor, increases the cytotoxicity of photodynamic therapy (PDT) on human glioblastoma (U87) and (U25ln) cells. U87 and U25ln glioblastoma cells were cultured and treated with Photofrin^®^-PDT as a photosensitizer. Tamoxifen significantly increases the activity of Photofrin^®^-PDT on U87 and U25ln human glioblastoma cells [83].

Photofrin^®^ (sodium porfimer) is a photosensitizer whose selective action results from a high affinity to lipoproteins, increased activity of the LDL receptor in cancer tissue and the absence or incompleteness of the lymphatic system in cancer tissue. This compound achieves its cytotoxic effect by oxidizing cellular components such as mitochondrial enzymes. In addition, Photofrin^®^ causes the destruction of tumor capillaries, which accelerates the death of cancer cells. The observed side effects are skin symptoms, but they are not severe [84].

#### 3.2.4. Hypericin

Photodynamic therapy (PDT) with Hypericin (HY) is gaining more and more interest as a potential treatment method for treating various tumors [85]. This compound shows high phototoxicity against malignant cells and accumulates to a greater extent in glioblastoma cells compared to neurons [86]. The incubation of cells for more than 3 h in a 100-fold dilution of Hypericin solution is the most effective for PDT, and the use of a low-intensity LED lamp leads to the highest rate of apoptosis [87]. Glioblastoma cells can be effectively inactivated by HY-PDT after short-term incubation and exposure to low doses of light. The results of the effect of HY-PDT on tumors are good and justify the further evaluation of HY-PDT in the treatment of malignant glioma [86]. One study observed that, in the short term, Hypericin-assisted PDT was not effective in large (greater than 1 cm^3^) tumors, but treatment significantly slowed tumor growth for tumors smaller than 1 cm^3^. Thus, it was found that PDT with Hypericin is effective in the treatment of remnants of small tumors [85]. Hypericin-PDT has been shown to be a promising therapeutic approach in the treatment of WHO IV glioblastoma [88]. In a study involving cell lines from patients with head and neck cancer, differences in response to PDT showed no correlation with photosensitizer uptake [89].

#### 3.2.5. Talaporfin

Talaporfin is a chlorin-based photosensitizer used in photodynamic therapy (PDT). The potential efficacy and safety of intraoperative photodynamic therapy (PDT) with Talaporfin sodium and 664 nm semiconductor laser irradiation was investigated in patients with primary malignant brain parenchymal tumors. 

Cutaneous adverse reactions attributable to the administration of Talaporfin sodium occurred in 7.4% of patients and included rash, blisters and erythema. Skin photosensitivity test results were relatively mild and resolved completely within 15 days after photosensitizer administration in all patients. It was found that intraoperative PDT with the use of Talaporfin sodium and a semiconductor laser may be considered as a potentially effective and sufficiently safe option for the adjuvant treatment of primary malignant brain parenchymal tumors. The inclusion of intraoperative PDT in the strategy of combined treatment may have a positive effect on overall survival and local tumor control, especially in patients with newly diagnosed glioblastomas (GBM) [90]. The safety and efficacy of photodynamic therapy (PDT) with Talaporfin sodium was investigated in patients with surgically unresectable malignant gliomas that invaded areas of the brain related to language and motor functions. The subject of the study was another 14 adult patients with malignant gliomas, in whom the invasion of eloquent brain areas was found with preoperative imaging diagnostics. Of these, six patients had newly diagnosed tumors and eight patients had recurrent tumors. None of the patients experienced adverse events directly related to PDT. Light protection was only required for about 3 days after PDT. Photodynamic therapy as an adjunct to surgical resection allowed for better therapeutic results than conventional protocols, especially in patients with newly diagnosed malignant gliomas [91].

#### 3.2.6. Other Photosensitizers

This section describes other photosensitizers used in research on the treatment of brain tumors.

Carboranyl-containing chlorins have an affinity for tumors, low cytotoxicity under dark conditions and a strong absorption in the red region of the optical spectrum. Tetrakis(p-carboranylthio-tetrafluorophenyl)chlorin (TPFC) is a new synthetic chlorin containing high boron carboranyl. In an in vitro PDT assay, the cell survival fraction after laser irradiation (9 J/cm^2^) was 0.035. [92].

The ruthenium-based photosensitizer TLD-1433 with apotransferrin (Rutherrin) was tested in a rat glioblastoma model. In the case of Rutherrin, much lower absorbed energy was sufficient to achieve the LD50 compared to 5-ALA-PDT. This photosensitizer provides a higher rate of specific uptake in tumors compared to the normal brain. After a single treatment, a significant increase in survival was observed in glioblastoma rats with Rutherrin-mediated PDT compared to PpIX. Rutherrin-PDT also showed an increased infiltration of CD8^+^ T cells into tumors. Rutherrin-PDT was well tolerated, providing safe and effective treatment for RG-2 glioma [93].

The effect of Tetrahydroporphyrin-tetratosylat (THPTS-PDT) in combination with ionizing radiation (IR) on glioblastoma cells was investigated in vitro and in vivo. Tetrahydroporphyrin-tetratosylat-PDT significantly reduced proliferation, metabolic activity and clonogenic survival and induced cell death mainly through apoptosis and autophagy. Tetrahydroporphyrin-tetratosylat alone showed no toxicity without irradiation. This study demonstrated the effective action of THPTS-PDT on glioblastoma cells, both in vitro and in vivo [94].

The use of PDT in the treatment of GBM was proposed by using chlor-aluminum phthalocyanine (AlClPc) encapsulated in a new drug delivery system (DDS) designed as a nanoemulsion (AlClPc/NE). Study results suggest that AlClPc/NE-PDT induces cell death in U87 MG glioblastoma cells in a dose-dependent manner and therefore may serve as an effective adjuvant therapy in malignant glioma. Chlor-aluminum phthalocyanine NE-PDT uses a low dose of visible light and can be used in conjunction with other classic GBM treatments, such as a combination of chemotherapy and surgery [95,96].

Photodithazine is a chlorin being evaluated for its effectiveness in PDT for glioblastomas. In the analysis of experiments using PDZ, 100% cell death was found at various concentrations of PDZ [97]. Glioblastoma cell viability assays 9 L/lacZ showed a reduction in the number of viable cells after PDT using Photodithazine. Reactive oxygen species production was dependent on the photosensitizer concentration. Photodithazine turned out to be an interesting photosensitizer in the treatment of glioblastoma [98].

(3S,4S)-14-ethyl-9-(hydroxymethyl)-4,8,13,18-tetramethyl-20-oxo-3-phorbinepropanoic acid (ETPA) is the main metabolite of the North Pacific echinoderm Ophiura sarsii. As a chlorin, ETPA efficiently generates singlet oxygen upon photoactivation with red light and exhibits strong submicromolar phototoxicity against a panel of in vitro tumor cell lines. In a mouse glioblastoma model, an intravenous injection of ETPA combined with targeted red laser irradiation induced strong necrotic ablation of the brain tumor [99].

2-[1-hexylethyl]-2-devinylpyruvate alpha (HPPH or Photochlor) is a photosensitizer being evaluated for use in the treatment of malignant gliomas with PDT. Twenty-four hours after an injection of 0.5 mg/kg HPPH, the ratio of drug in the tumor to the brain ranged from 5:1 to 15:1. Increased survival was observed in each of the groups of animals treated with HPPH-PDT. These data suggest that HPPH may be a useful adjuvant in the treatment of malignant gliomas [100].

Pheophorbide A (Ph-A), a photosensitizer of low dark toxicity, is activated by a Q-switched acoustically neodymium-yttrium-argon (Nd:YAG) laser that achieves deep tissue penetration. In vivo PDT studies using T9 glioma cells implanted in the dorsal region of F344 rats showed tumor eradication in four out of six rats. The combination of PDT and laser hyperthermia resulted in tumor eradication in all six rats. According to the study, the combination of PDT and hyperthermia is a promising method of treating tumors [101].

The dye IR-780 (IR780) and tube-forming peptoids (PepIR) were synthesized and self-assembled into crystalline nanotubes (PepIR nanotubes). PepIR nanotubes showed excellent performance in PDT/PTT. In addition, the efficient loading of doxorubicin (DOX) was achieved by the large surface area of the nanotubes and contributed to effective and synergistic chemotherapy against glioblastoma cells. Due to the unique properties of peptoids and peptoid nanotubes, the DOX-loaded multimodal PepIR nanotubes developed in this work hold great promise for the future therapy of glioblastoma in the clinic [102].

The new promising photosensitizer PDT SIM01 was evaluated in an orthotopic C6 tumor model in rats by comparison with HPD and *m*-THPC. The optimal concentration was found after 12 h for SIM01, 24 h for HPD and 48 h for *m*-THPC. The most favorable normal tissue/cancer ratio was found after 12 h for SIM01 and 48 h for HPD and *m*-THPC. The average survival of rats treated 12 or 24 h after SIM01 injection was significantly better compared to the control, HPD- or *m*-THPC groups. The results of the study confirm that SIM01 is as effective as *m*-THPC but has much more favorable pharmacokinetics [103].

A new photosensitizer, ATX-S10.Na(II), has been investigated for possible use in photodynamic therapy (PDT) for glioblastoma. Cytotoxicity was found to be dependent on both drug concentration and laser energy. The concentration of ATX-S10.Na(II) in Fischer rat brain tumors peaked 2 h after administration, and the tumor/normal brain concentration ratio was as high as 131 at 8 h. Intratumoral PDT for irradiated intracranial tumors showed an antitumor effect without serious side effects [104].

ZnPcS4-BSA is a newly synthesized photosensitizer that has beneficial properties against U251 glioblastoma cells. The results of a study indicate that the uptake of ZnPcS4-BSA by tumor cells reaches its maximum after incubation for 4 h. This compound has no significant effect on cell survival without light irradiation. After using a laser of 150 J/cm^2^, it was found that cell inhibition indices increase with the concentration of ZnPcS4-BSA. The rate of cell apoptosis after PDT was significantly higher than in the control group. At the same time, after using PDT, the expression of VEGF in cancer cells increases 5.6 times. Photodynamic therapy based on ZnPcS4-BSA can induce effective apoptosis [105,106].

Hydrophilic nanoparticles of polyethylene glycol (PEG)-chlorin e6 (Ce6) chelated with a gadolinium ion (Gd3^+^) (PEG-Ce6-Gd NPs) were synthesized through the process of chelation and self-assembly. Studies have shown the lack of toxicity of this compound to cancer cells without irradiation and a significant reduction in the weight and size of mouse brain tumors after laser irradiation. PEG-Ce6-Gd NPs have great potential in the diagnosis and PDT treatment of gliomas [107].

To improve the effectiveness of GBM therapy, a new strategy for photosensitizer delivery was developed by using ‘photo-controlled platelets.’ It involves the use of platelets as carriers of the photosensitizer to the tumor. In this study, a nanocomposite (BNPD-Ce6) consisting of chlorin e6 (Ce6) loaded into boron nitride nanoparticles with a surface coating of polyglycerol and doxorubicin was developed. In the study, mouse platelets were loaded and BNPD-Ce6@Plt was obtained. Laser irradiation with a wavelength of 808 nm induced ROS generation in BNPD-Ce6@Plt, which showed the rapid activation, aggregation and release of BNPD-Ce6 into cocultured mouse GBM GL261 cells, which in turn showed marked ROS generation, DNA damage with reduced cell viability and death. There was no obvious tissue damage in the vital organs. The results of this study demonstrate that platelets can act as effective carriers that deliver photosensitizers in a photo-controlled manner in GBM therapy [108].

New compounds containing asymmetrically substituted phthalocyanines were synthesized, including Zn(II)Pc1, which turned out to be a very efficient singlet oxygen generator and a promising photosensitizer for PDT applications. Biodistribution studies revealed that radiolabeled Zn(II)Pc1 showed significant uptake in the brain, intestine, pancreas and ovary. Hence, these Pcs derivatives could also be promising candidates for the nuclear imaging of tumors [109].

A nanoprotein (Nanobody) was developed that binds to the extracellular side of the viral G protein-coupled receptor US28, which is detected in glioblastomas. The nanoprotein was coupled with the water-soluble photosensitizer IRDye700DX. This conjugate selectively killed US28-expressing glioma cells in 2D and 3D cultures when irradiated with near-infrared light. These data provide a new perspective on the use of this large family of receptors for targeted therapies [110].

Significant cytotoxicity was observed in glioblastoma cells during the irradiation of LaF3:Tb nanoparticles combined with the photosensitizer *meso*-tetra(4-carboxyphenyl)porphyrin (MTCP). These particles are characterized by good dispersion in aqueous solutions and a high biocompatibility [111].

Porphyrazine derivatives (bp I–IV) showed accumulation in neuronal and glioblastoma cells, but their rates of internalization, subcellular localization and toxicity in the dark differed significantly. Porphyrazine II was the most promising photosensitizer. It effectively killed glioblastoma cells while remaining nontoxic to primary neuronal cells [112]. Studies have shown that the use of pz I–IV leads to a significant decrease in the main calcium functional parameters of neuronal–glial networks and causes significant changes in the characteristics of the network. The observed negative effects of pz I–IV intensified under the influence of PDT. Considering the significant restructuring of the functional architecture of neural–glial networks, which can lead to serious disorders of synaptic transmission and loss of brain function, as well as the possibility of the direct application of PDT based on pz I–IV in the therapy of brain tumors, it is highly controversial. Nevertheless, the unique properties of pz I–IV retain a great prospect of their use in therapy for tumors of other origins and cellular metabolism [113].

Ce6-AuNP-Lf is a potent phototherapeutic nanoconjugate that consists of gold nanoparticles (AuNPs) and photosensitizers (PSs) prepared by disulfide conjugation between chlorin e6 (Ce6) and glutathione-coated AuNPs. PEGylated lactoferrin (Lf-PEG) was incorporated into the surface of AuNPs to allow for oral administration and targeting of the nanoconjugate to glioblastoma multiforme (GBM) cells. The engineered nanoconjugates significantly improved ROS generation, allowing sufficient PDT for this tumor. Thanks to the conjugation of the nanoconjugate with Lf, effective targeting of the agent to tumor cells was achieved. These results suggest that Ce6-AuNP-Lf is a potent phototherapeutic GBM nanoconjugate that can be administered orally [114]. Table 2 presents a summary of the most commonly used photosensitizers in PDT in the treatment of brain tumors.

### 3.3. Third Generation Photosensitizers

Third-generation photosensitizers are innovative composites, functionalized nanostructures and technologies that enable more effective drug delivery to neoplastic lesions [115]. Currently, there are many possibilities to create these types of photosensitizers, and the list of strategies for developing newer constructs is constantly expanding. 

Third-generation photosensitizers are the synthesis of second-generation photosensitizers with groups such as peptides, antibodies, carbohydrates and amino acids. Another example is the creation of a carrier, e.g., in the form of micelles or liposomes as a transport medium for the applied photosensitizer. Mfouo-Tynga et al. [115] reviewed the most important features of third-generation photosensitizers in PDT. The most common components that combine with photosensitizers are monoclonal antibodies [116], saccharides [117], nanoparticles [118], hyaluronic acid [119], liposomes [120], polymer micelles as well as small molecules and inhibitors [115].

Third-generation photosensitizers are being tested in both clinical and preclinical trials. Their characteristic feature is that they have increased selectivity for cancer cells [121]. Currently, there are many documented studies in which newly designed composites of third-generation photosensitizers were used. Some published literature reports are presented below. 

Ibarra et al. [122] in their study, evaluated the strategy of delivering polymer nanoparticles on a monocyte carrier to improve the effectiveness of PDT in the treatment of glioblastoma. To this end, they used a carrier of human monocyte cells and mouse monocytes from bone marrow as a composite for easier penetration. The results of the experiment were as follows: No effect of polymer nanoparticles on monocyte viability in the absence of light was observed. The effectiveness of the therapy carried out in vitro was higher with the use of monocytes as a carrier compared to the therapy that used polymer nanostructures without a carrier. The authors confirm that the use of monocytes as carriers for polymer nanoparticles increases the effectiveness of PDT [122].

Caverzán et al. [123] in turn, evaluated the effect of PDT in combination with conjugated polymer nanoparticles on glioblastoma cells. The aim of the experiment was to compare the PDT activity supported by polymer nanoparticles on three glioblastoma cell lines with different initial contents of reactive oxygen species. Three human glioblastoma cell lines (U-87 MG, M059K and T98G) of male origin were used in this study. Polymer nanoparticles were developed by using a fluorescent semiconductor polymer. The cellular uptake of polymer nanoparticles was assessed by flow cytometry. The results of the study were as follows: cells from the T98G line were the most resistant to PDT treatment with polymer nanoparticles in comparison to the cells from the MO59K line and from the U-87 MG line. The initial content of antioxidant enzymes is a key feature of glioblastoma cells. Their association with polymer nanoparticles may be crucial to designing more effective methods and therapies based on nanoparticles [123]. 

Another example of the use of third-generation photosensitizers is the research conducted by Ibarra et al. [124]. The authors used conjugated polymer nanoparticles doped with porphyrin to treat brain and colorectal cancer cells. The aim of this study was to assess the biocompatibility of the PDT mechanism supported by porphyrin-doped polymer nanoparticles on different cell lines (98G, SW480 and RAW 264.7). In the results of the study, the authors confirmed that PDT was effective for all three cell lines. Oxidative stress was observed, which in turn led to cell apoptosis [124]. 

The last literature report cited in the field of PDT supported by conjugated polymer nanoparticles for the treatment of glioblastoma cells is the article by Caverzán et al. [125] from 2023. In this experiment, the authors, starting in 2020, used three glioblastoma cell lines and PDT coupled with polymer nanoparticles. In addition, the authors used metronomic photodynamic therapy, which involves the administration of low-intensity light for a long period of time. This is one of the alternative therapeutic methods that fills the limitations of standard and commonly implemented PDT schemes. In the experiment, the authors used different methods of irradiation in different fluence coefficient ranges. The results of the experiment confirmed that metronomic photodynamic therapy initiated the death of cancer cells already at very low concentrations of polymer nanoparticles. The specificity of irradiation generated the so-called photokilling in all glioblastoma cell lines initiating various mechanisms of cell death. This experiment provides information on the development of advanced PDT concepts in conjunction with the application of lower irradiance. The polymer nanoparticles used contribute to the inhibition of tumor growth and initiate subsequent cell death pathways [125]. 

### 3.4. Limitations of Photosensitizers

One of the main limitations of photosensitizers (especially in brain tumors) is their low solubility in water. As a consequence, photosensitizers have poor penetration and permeability in tumor tissues and cells. According to Sun et al., photosensitizers of the first and second generation have low effectiveness in the treatment of cancer cells, which makes their accumulation in the tumor limited and less effective [126,127]. 

In addition, first-generation photosensitizers have a long half-life lasting from several days to even several weeks [127]. Most of the photosensitizers used have an absorption range of 400 to 700 nm. The penetration of this type of light in tissues is limited, resulting in reduced effectiveness. Second-generation photosensitizers have better photostability than first-generation photosensitizers. In addition, they absorb light of longer wavelengths, which have the ability to penetrate deeper into tissue. According to Udrea et al. [127] the main disadvantage of second-generation photosensitizers is their localization in cancer cells and poor water solubility, which limits the intravenous application of these photosensitizers. 

The solution to certain limitations and difficulties related to limited penetration or lack of light delivery is the use of third-generation photosensitizers and composites or platforms based on nanomedicine.

### 3.5. Optical Characteristics of Photosensitizers

According to Ormond and Freeman, a good and effective photosensitizer should have features such as a pure chemical composition (without unnecessary admixtures or fixatives), easy to obtain from generally available precursors, a high quantum efficiency of singlet oxygen, an absorption range in the range of 680–800 nm with a high extinction coefficient (ε max), characterized by effective accumulation in cancer cells and tissues, characterized by low toxicity in the dark in the absence of light, easy to apply, well soluble in body fluids and easily removed from the body [128]. With regard to the photosensitizers discussed herein, 5-ALA has an extinction coefficient of 5000 M^−1^ cm^−1^ and a singlet oxygen quantum yield of 0.56. Temoporfin has an extinction coefficient of 35,000 M^−1^ cm^−1^ and a singlet oxygen quantum yield of 0.87. Photofrin has an extinction coefficient of 3000 M^−1^ cm^−1^ and a singlet oxygen quantum yield of 0.89. Talaporfin has an extinction coefficient of 40,000 M^−1^ cm^−1^ and a singlet oxygen quantum yield of 0.77. Hypericin has an extinction coefficient of 44,000 M^−1^ cm^−1^ [128]. 

### 3.6. Irradiation Conditions

According to Quirk et al. [129] standardized guidelines for treatment protocols with PDT are still lacking. The main parameters such as the dose, wavelength of laser light, method of light delivery and selection of the appropriate photosensitizer depends on the type and location of the disease. The selection of the parameters affects the effectiveness of the treatment [129]. Initially, the light sources used were argon lasers. In turn, diode lasers were introduced into clinical practice at the beginning of the 21st century. A few years ago, light-emitting diodes (LEDs) were introduced to the treatment as an innovative and, importantly, less expensive way of delivering light. Applied LEDs enable higher light output and limited spectral characteristics. The problem of scattered light has been solved by using appropriately dedicated optical fibers. Their cylindrical tips are ideal for interstitial photodynamic therapy commonly used in the treatment of brain tumors. The stereotactic distribution of fibers in brain tissue during PDT improves the treatment efficacy. Another way of applying light is to use and encapsulate the light in a balloon, which is filled with a diluted liquid photodistributor. This allows the light to be evenly distributed over the entire surface of the balloon. Another way is to continuously irrigate the resection area with a photodistributor. The main advantage of this method is the reduction in heat generated during therapy. In addition, the reduction in plasma and blood accumulation in the cavity enhances the light distribution during treatment. 

Under in vitro conditions, providing light is much simpler compared to clinical trials. For example, an in vitro study was described by Vilchez et al. [44] who treated human glioblastoma cells. For the treatment of PDT, they irradiated the cells by using a monochromatic light source with a wavelength of 635 ± 17 nm. They used a system of LED diodes. They monitored the irradiation intensity by using a power meter [44]. Another example of in vitro research is the work of Kamoshima et al. [46]. They also used glioblastoma cell lines. In their research, they used a diode laser with the following parameters: 635 ± 5 nm, 5–100 mW/cm^2^ and a total light dose of 2.5–50 J/cm^2^. It is worth noting that all steps of the PDT protocol were performed by the authors in dark conditions. In turn, Yi et al., who conducted research on rats, used a helium–neon laser with a wavelength of 632.8 nm [45]. Hirschberg et al. [62] also conducted studies on rats that were immobilized in a stereotactic frame. Quartz fiber was inserted through the incised skin directly interstitial into the brain. The wavelength of the laser light was 632 nm. The irradiation time was 45 min or 90 min. Kimura et al., also using a stereotactic frame, irradiated the right lateral skull of a rat with LED light from a distance of 3 cm above the skull [61]. Fisher et al. delivered light (635 nm wavelength and 24 J energy) by using an isotropic emitter, which was inserted 1 mm below the dura mater in the upper part of the tumor [57]. On the other hand, in an in vivo study in which tests were carried out on patients diagnosed with a brain tumor, intraoperative PDT is more complicated compared to in vitro studies. An example of a work is the work by Muller and Wilson. In order to diffuse light into the tumor cavity, an inflatable balloon was used which was filled with a diluted Intralipid. Thus, the surface of the balloon was almost coplanar with the surface of the tumor. The liquid applied ensured uniform irradiation over the entire surface of the balloon [75]. A similar study design was conducted by Aziz et al. [81]. The light was applied to the excision site, which was filled with a balloon. A diode laser with a wavelength of 630 nm was used. The first application was carried out in the recovery room, and another one was carried out at the bedside in the ward [81]. Muragaki et al. [90] in turn, conducted a study on treating patients with malignant brain tumors with PDT. In their study, light was applied to the resection cavity after tumor excision. A semiconductor laser with a wavelength of 664 nm and a diameter of 1.5 cm was used. In particular, areas where metastasis or recurrence of the disease could have occurred were taken into account, avoiding irradiation of the same area twice [90]. Akimoto et al. [91] used an optical navigation system and electrophysiological monitoring to deliver the laser light, leaving the tumor bed area to be exposed to the laser light. The treatment surface area was 1 cm^2^. In the experiment, they used a laser with a wavelength of 664 nm, and the exposure time was 180 s [91].

### 3.7. Light-Delivery Systems

One of the innovative light-delivery systems are implantable devices that enable light delivery during PDT. Another example of enhancing the effectiveness of therapy using light sources is lasers in the near-infrared range, which allow tissue penetration up to 3 cm. Thanks to this, the light reaches the interior tumor more precisely [130].

According to Cramer and Chen, the most preferred source of light in PDT is lasers with a longer wavelength, which penetrates deeper into the tumor site, delivering photons of sufficiently high energy to activate photosensitizer molecules. As mentioned earlier, PDT typically uses lasers with wavelengths ranging from 400 to 900 nm, with the most common range being 600–800 nm. The application of light can be continuous or pulsed. The latter enables the oxygenation of the tumor at regular intervals [121].

According to Yoon et al. [131] in order for the light to be delivered precisely inside the tumor, the light source must be delivered by using appropriate fiber optic devices. They usually consist of quartz fibers with cylindrical tips. Another type is optical fibers with a lens. These above-mentioned devices provide adequate dosimetry and high efficiency with little damage to normal tissue [131]. In order to minimize damage to normal tissue, a computer-controlled pulse delivery system is practiced while photosensitizers are applied directly into the artery. Such combinations are practiced in cases of prostate cancer. For brain tumors, near-infrared upconversion nanoparticles and bioluminescence are practiced.

The intraoperative treatment of brain tumors with PDT by using various photosensitizers was initiated in the 1990s. The first photosensitizer used was Photofrin^®^. The research group consisted of 56 patients with recurrent supratentorial gliomas [132]. These were patients who had previously undergone ineffective radiotherapy. The results of the experiment clearly indicated that patients who received intraoperative PDT treatment lived longer than patients who received surgical treatment alone. In recent years, PDT has established itself as a safe and selective method that extends and improves the quality of life of patients with brain gliomas. Trials are currently underway to use metronomic photodynamic therapy (a new strategy involving the use of lower doses of PDT, but over a longer period of time) analogous to metronomic chemotherapy in the treatment of brain tumors. Both methods are verified with molecular and clinical tests.

One of the latest studies on intraoperative photodynamic therapy is the work by Vermandel et al. [133] in which the authors developed a pilot study in the field of intraoperative PDT in combination with 5-ALA in the treatment of glioblastoma. The main objective of the study was to evaluate the efficacy and safety of intraoperative PDT for the treatment of glioblastomas. The results confirmed that this type of therapy in combination with 5-ALA is effective, but it requires further evaluation and analysis, mainly with the participation of a larger number of patients [133]. 

Another example is the work by Hirschber et al. [134] in which the authors reviewed the effect of intraoperative PDT on malignant brain tumor cells, both in vitro and in vivo. The results confirmed that PDT repeated several times at long intervals is more effective compared to the standard single treatment [134].

## 4. Limitations of this Study

The main limitation of this study is the risk of bias, including bias at the stage of the selection of review and research articles and bias resulting from previously published works. Another limitation was inconsistency and a lack of precision, which may have resulted in shortcomings in the drafting of this review. In addition, the selection of only some photosensitizers from the first and second generation (supplemented by the third generation) is a certain limitation and narrows down the selection criteria.

## 5. Conclusions

Brain tumors are a specific group of oncological processes in which the location and nature of the growth are of key importance for clinical symptoms and prognosis. The surgical treatment of tumors of the nervous system, unlike other oncological processes, usually cannot be carried out in accordance with the principle of oncological purity, i.e., the removal of the proliferative process along with the margin of the surrounding tissues; the very specific nature of the nervous tissue of the brain does not allow this. One of the innovative methods of treating brain tumors is photodynamic therapy. This paper presents an overview of the most commonly used photosensitizers in photodynamic therapy for the treatment of brain tumors. The most commonly used photosensitizers include 5-aminolevulinic acid, Temoporfin—THPC, Photofrin^®^, Hypericin and Talaporfin. In turn, third-generation photosensitizers are innovative composites, nanostructures and technologies that enable the more effective delivery of drugs to neoplastic lesions. Currently, there are many possibilities to create this type of photosensitizer, and the list of development strategies for newer and newer models is constantly expanding. There are many challenges in treating tumors with PDT. One of them is the way of delivering light, limiting photosensitizers and their removal from organisms. Based on this review, it can be concluded that there are many solutions starting from the LEDs used to optical fibers and the nanoparticles that facilitate the delivery of light to the inside of the tumor. The effectiveness of PDT is high, but research is still underway to improve it. In order to improve the effectiveness of PDT for the treatment of brain tumors, it is necessary to conduct further studies, both in vitro and in vivo, which will enable the selection of an appropriate treatment protocol.

## Figures and Tables

**Table 1:** Inclusion and exclusion criteria for review.

*Inclusion*
The analyzed cases were brain tumors
Both in vivo and in vitro studies were included
Both review articles and research articles were included
Studies in which the research groups were adult patients were included in this review
This review included papers in which such photosensitizers were characterized as 5-ALA, Temoporfin, Photofrin, Hypericin or Talaporfin
Recent papers describing other types of photosensitizers and third-generation photosensitizers were also included
***Exclusion***
Articles in a language other than English or Polish
PDT for other types of cancer
Studies that used chemotherapy in combination with PDT were excluded
Studies in which magnetic resonance imaging, computed tomography or other tools were used for diagnostics were also excluded
Studies conducted on pregnant women or children
Analysis of the immune and anti-inflammatory response after PDT

**Table 2:** A summary of the most commonly used photosensitizers in PDT for the treatment of brain tumors.

No.	A Type of Photosensitizer	Structure	The Wavelength of the Light Source (nm)	Characteristics/ Application	Potential Side Effects	References
**1.**	5-ALA	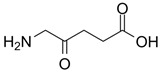	635	Possible intraoperative fluorescent guidance, increase in PpIX accumulation, treatment of malignant gliomas	Minimal skin sensitization	[38,39,40,41]
**2.**	Temoporfin —THPC	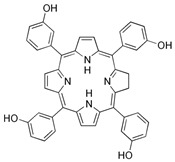	650	Squamous cell tumors of the head and neck	May cause complaints associated with high photosensitivity of the skin	[70,71]
**3.**	Photofrin	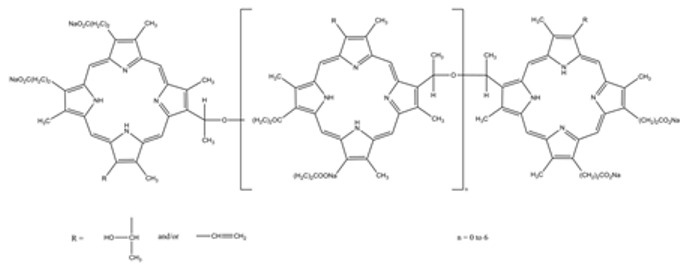	630–660	Inhibits the invasiveness of glioblastoma cells; has the ability to induce VEGF expression in the tissue adjacent to the tumor; causes a transient increase in cell proliferation associated with strong activation of astrocytes and microglia in the treated region	Slight skin irritation may occur	[76]
**4.**	Hypericin	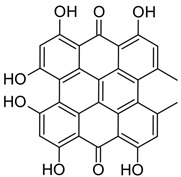	590–660	Shows a promising therapeutic approach in the treatment of glioblastoma	Systemic side effects in healthy tissues	[88]
**5.**	Talaporfin	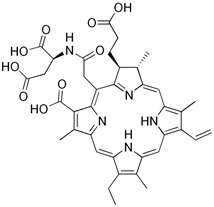	630–667	Treatment of primary malignant brain parenchymal tumors	The possibility of side effects on the skin (rash, blisters, erythema)	[90]

## Data Availability

All data have been included.
